# Psoas Abscess Formation in Suboptimally Controlled Diabetes Mellitus

**DOI:** 10.1155/2011/249325

**Published:** 2011-07-28

**Authors:** A. J. Lansdown, A. Downing, A. W. Roberts, D. Martin

**Affiliations:** ^1^Department of Integrated Medicine, University Hospital of Wales, Heath Park, Cardiff CF14 4XW, UK; ^2^Department of Radiology, University Hospital of Wales, Heath Park, Cardiff CF14 4XW, UK

## Abstract

Psoas abscess formation is a rare entity for which diabetes mellitus remains a major predisposing factor. Diabetes has long been associated with a predisposition to unusual and more serious infections. Here we present two cases that demonstrate that chronically suboptimally controlled diabetes remains an important marker for the development of primary psoas abscess. It is important to include psoas abscess in the differential in such patients to ensure early diagnosis and treatment.

## 1. Introduction

Psoas abscess formation is a rare entity that can prove a diagnostic challenge. Patients with diabetes mellitus are more susceptible to infections and in particular unusual infections caused by rare organisms [1]. Here we present two cases, both of patients with poorly controlled diabetes mellitus, who develop the complication of psoas abscess.

## 2. Case Report A

A 27-year-old woman, with a 13-year history of poorly controlled diabetes mellitus, presented to the emergency department with a 3-week history of right flank, lower back and hip pain, difficulty in weight-bearing, and rigors. There was no history of trauma and she denied any dysuria or vaginal discharge. Her past medical history included asthma and a previous appendicectomy.

On examination, she had a fever and tachycardia. She preferred to sit upright with right hip flexed and abducted. There was tenderness over the right sacroiliac joint, pain on straight right leg raising at 30 degrees but no abdominal tenderness. Full blood count and biochemistry revealed a leukocytosis of 14.9 × 10^9^/L, C-reactive protein of 268 mg/L, albumin 19 g/L, HbA1c 16.3%, and normal renal function. Urine culture yielded staphylococcus aureus. Pelvic X-ray was unremarkable.

**Figure 1:**
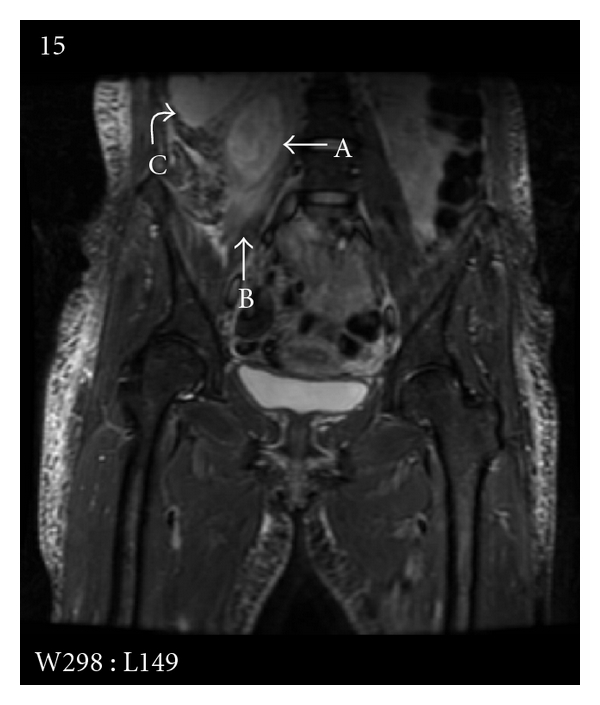
MRI, coronal view. Right-sided psoas abscess (A) with surrounding oedema (B) pushing the right kidney anterolaterally (C).

An ultrasound scan of the renal tract showed mild hydronephrosis of the right kidney with proximal hydroureter. Subsequent magnetic resonance imaging (MRI) confirmed a right psoas abscess extending from the level of the first lumbar vertebra to the first sacral vertebra, pushing the right kidney anterolaterally with some hydronephrosis (Figure 1). There was no evidence of osteomyelitis.

A percutaneous drain was positioned under ultrasound guidance into the psoas abscess. Staphylococcus aureus was grown from the pus, and the patient was successfully treated with appropriate antibiotics.

## 3. Case Report B

A 43-year-old man was referred to the acute medical unit with a 7-week history of back pain. He had a history of diabetes mellitus for 10 years, with evidence of peripheral diabetic neuropathy and retinopathy. A spinal cord stimulator was insitu for his neuropathy and he had previous multiple admissions with diabetic ketoacidosis. Recently he had received several courses of antibiotics in the community for pyrexia of unknown cause.

On examination, there was fever and marked tenderness of the right flank. Full blood count showed a normocytic anaemia, neutrophilia of 25.82 × 10^9^/L, C-reactive protein 384 mg/L, glucose 19.5 mmol/L, creatinine 129 umol/L, and venous bicarbonate of 13 mmol/L. Most recent HbA1c was 14.3%. Chest and abdominal X-rays were normal. Urine specimen was unremarkable.

**Figure 2:**
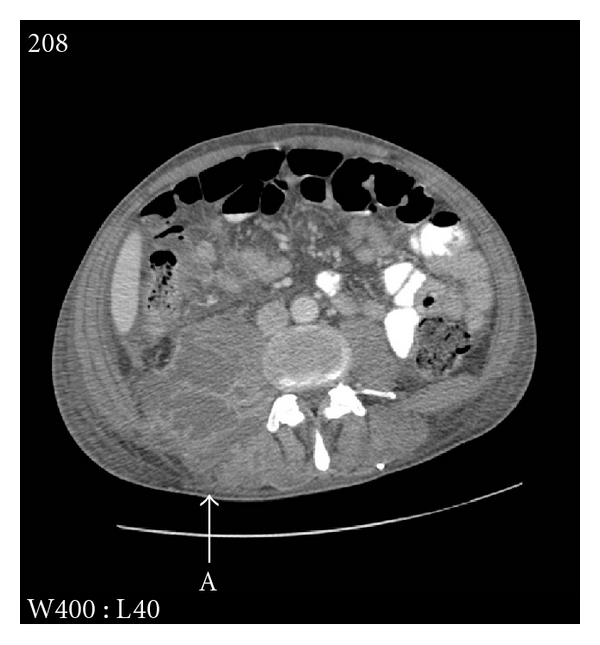
CT, axial view. The loculated right-sided psoas abscess extends to the skin surface (A).

A computed tomography (CT) scan of abdomen and pelvis was subsequently performed. This confirmed a complex multiloculated right psoas abscess, extending to the skin surface posteriorly (Figure 2). No bony or renal tract abnormalities were noted. There was no evidence of infection at the spinal stimulator site. 

Given the loculated nature of the abscess, it was drained surgically and yielded a moderate growth of staphylococcus aureus. The patient was treated appropriately for his diabetic ketoacidosis, including antibiotic therapy for the psoas abscess.

## 4. Discussion

Psoas abscess is a rare entity. The worldwide incidence was noted to be 12 cases per year 100,000 in 1992, but the current incidence is unknown, although it is thought to be on the increase [2, 3]. Psoas abscesses can be arbitrarily divided into primary or secondary depending on whether a separate focus can be identified. The most common predisposing factors for primary abscesses are intravenous drug use and diabetes mellitus [4]. Osteomyelitis, discitis, and genitourinary tract infections remain the main causes resulting in secondary psoas abscess formation, with bowel infections and trauma appearing less common than previously reported [2, 5–8]

Both our cases demonstrate poorly controlled diabetes as being the primary factor resulting in abscess formation, and we would suggest it should be a marker to raise the index of suspicion in those cases who present acutely unwell. Indeed, chronically poorly controlled diabetes has long been associated with a predisposition to unusual and more serious infections [1]. It could be argued that Case A was secondary to a complicated urinary tract infection, although it seems more likely the hydronephrosis and urinary culture of staphylococcus aureus were a direct result from the primary abscess. Likewise, Case B points towards poor glycaemic control as being the primary causative factor rather than it being secondary to another source of infection.

The presentation of psoas abscess is classically a triad of fever, flank pain, and limitation of hip movement, although previous case series have shown that all three are found only in a minority. Our cases demonstrate the importance of a positive psoas sign in making the diagnosis of psoas abscess, with the presence of worsening pain on hip flexion indicating irritation to the iliopsoas group of hip flexors in the abdomen [8, 9]. A high level of suspicion for diagnosis is required, particularly in the context of underlying predisposing conditions.

The most common causative organism associated with primary psoas abscess is staphylococcus aureus [4, 5, 7, 8] and both our cases support this finding. Escherichia coli is the leading cause of secondary abscess formation, although Mycobacterium tuberculosis is also an increasingly important pathogen in certain areas of the world and in the immunocompromised [4].

CT scanning is probably the standard imaging technique of diagnosis of psoas abscess. MRI and ultrasound are also valid methods, although the latter can result in false-negative results [6–9].

It has been suggested that the initial management of psoas abscess should be nonsurgical, with small abscesses treated with antibiotics alone and reserving surgery for more complicated reoccurrences [10]. The underlying cause of the abscess will, of course, help to determine the nature of intervention.

## 5. Conclusions

Both our cases demonstrate that chronically poorly controlled diabetes mellitus remains an important marker for the development of primary psoas abscess. It is important to include psoas abscess in the differential in such patients to ensure early diagnosis and treatment.

## 6. Learning Points

- Psoas abscess is a rare entity but thought to be on the increase.
- Poorly controlled diabetes mellitus is a risk factor for any complicated infection but is a notable predisposition for primary psoas abscess formation.
- Bone and genitourinary infections are the leading contributors to secondary abscess formation.
- Most patients present with fever, flank/hip pain, and limited hip movement, but few with the classic triad of symptoms.
- Staphylococcus aureus remains the most common pathogen in primary abscess formation.
- Antibiotic therapy and percutaneous drainage is the main treatment strategy, although more complicated recurrent cases may require surgical intervention.
